# Encoding of semantic structure shapes temporal order memory for visual object stimuli

**DOI:** 10.1007/s00426-025-02222-0

**Published:** 2026-01-13

**Authors:** Henry David Soldan, Carina Zoellner, Nora Alicia Herweg, Nurten Genc, Oliver Tobias Wolf, Christian Josef Merz

**Affiliations:** 1 Department of Cognitive Psychology, Institute of Cognitive Neuroscience, Faculty of Psychology, Ruhr University Bochum, Universitätsstr. 150, 44801 Bochum, Germany; 2 Department of Neuropsychology, Institute of Cognitive Neuroscience, Faculty of Psychology, Ruhr University Bochum, 44801 Bochum, Germany

**Keywords:** Generative episodic memory, Memory retrieval, Prior knowledge, Scenario construction, Semantic memory

## Abstract

Episodic memory does not perfectly reproduce past experiences but combines encoded episode-specific information and semantic knowledge in a constructive way. Previous research has shown that semantic category knowledge can bias location memory for individual items, suggesting that similar mechanisms may affect other key dimensions of episodic memory. Here, we investigated whether immediate temporal order memory is influenced by semantic relatedness between encoded items and whether this effect is modulated by semantic structure at encoding, episodic association strength and semantic typicality. Across two experiments, participants completed a temporal order memory task in which they encoded sequences of object images and subsequently judged the relative temporal proximity between items. Results showed that participants who encoded semantically structured sequences performed significantly better on congruent retrieval trials where the correct choice (the temporally closer item) was semantically related to the cue versus on incongruent trials where the incorrect choice was semantically related to the cue. This semantic congruence effect was stronger with shorter temporal distance between the cue and target item at encoding. Participants who did not encode semantically structured sequences did not show the semantic congruence effect. Overall, these findings demonstrate that semantic relatedness between encoded items can bias immediate temporal order memory depending on the presence of semantic structure within encoded item sets. We discuss these results as evidence that semantic knowledge influences temporal order memory through encoding of structured context, highlighting the alignment between semantic and temporal associations as an important modulating factor for this interaction.

## Significance statement

The episodic memory system reconstructs past experiences relying on episode-specific information and semantic knowledge. Across two experiments we show that semantic category knowledge influences memory for the temporal order of events: After encoding sequences of object images which were temporally clustered in categories, participants tended to retrieve semantically related items as having been presented closer in time. This was not the case when semantically related items were encoded in random order, suggesting a key role of semantic processing at encoding.

## Introduction

When recalling events from their personal past, humans rely on episodic memory to retrieve information about what they experienced in a specific spatiotemporal context. While episodic memory serves an important adaptive purpose, it does not always accurately reflect past events but tends to produce systematic errors that reflect its constructive nature (Schacter, 2012). The notion that episodic memory is inherently constructive entails that it recombines information from multiple sources to generate coherent scenarios and to enable decisions. Semantic knowledge is a key component of constructive memory processes and has been theorized to shape memory retrieval for a long time (Bartlett, 1932). For instance, classic work using the Deese–Roediger–McDermott paradigm (Roediger & McDermott, 1995) has demonstrated that semantic associations between items in studied word lists can result in false memories for items that were never part of the learned material. More recent evidence suggests that semantic and episodic memory are closely interconnected in terms of cognitive processes and underlying neural mechanisms (Renoult et al., 2019). However, how and under which conditions semantic knowledge systematically shapes episodic memory retrieval is still not completely understood. Thus, we aimed at extending this line of research by investigating how semantic knowledge impacts memory for the temporal order of event sequences.

The Scenario Construction Model (Cheng et al., 2016) postulates that episode-specific information is flexibly integrated with semantic knowledge to meet current information processing demands. More specifically, the model posits that episode-specific information is typically stored in the form of a memory trace representing the gist of an episode (i.e., its central aspects and structure). This episodic gist representation is then integrated and completed with consolidated semantic information to form a coherent scenario in a generative memory retrieval process (Cheng et al., 2016). Accordingly, memory retrieval should reflect both, episode-specific information and semantic knowledge, in situations where such generative memory processes are evoked. By extension, since most experience involves semantically structured event sequences (such as the temporal and spatial co-occurrence of related “animal concepts” during a visit to the zoo (see Pathman et al., 2023), semantic processing at encoding is likely to influence the generation of event representations and subsequent generative retrieval (Addis, 2018).

Previous studies have demonstrated episodic-semantic interactions in various memory tasks. For example, participants reliably exhibit robust effects of temporal contiguity and semantic relatedness in free recall tasks (Healey, 2018; Howard & Kahana, 2002). Pairing word items with semantically related context material at encoding is associated with enhanced retrieval performance for such items, constituting a semantic congruence effect (Bein et al., 2015). Notably, this can also be accompanied by increased false recall of semantically related items (Packard et al., 2017). Moreover, retrieval of image locations was found to be enhanced when a given item had been encoded near a cluster of semantically related items versus in a random location, while semantic typicality predicted retrieval bias towards category clusters (Tompary & Thompson-Schill, 2021). Similar effects of semantic knowledge on memory for episodic features have been shown for a naturalistic episodic navigation task involving encoding of semantically congruent and incongruent object locations (Zöllner et al., 2022). In another recent study, the quality of episodic memory for scenes was negatively related to semantic bias in memory retrieval (Ramey et al., 2022). The negative effect of semantically incongruent placements on location memory retrieval was absent for scenes which participants were able to fully recollect, suggesting a more pronounced influence of semantic processing on memory retrieval in case of weak episodic representations. Collectively, these findings suggest that a semantically structured encoding context can bias retrieval towards semantic expectations, with this effect potentially being modulated by the strength of episodic memory representations.

Arguably, these effects are applicable to the temporal dimension of episodic memory. In addition to physical space, time is a defining context factor in episodic memory organization with specific events being mapped to a specific spatiotemporal context (Tulving, 2002). Both space and time are encoded by the hippocampal memory system which enables the organization of discrete events in memory along spatial as well as temporal axes (Ekstrom & Ranganath, 2018). Early work has shown that spacing judgments for words in a study list are strongly influenced by whether items come from the same category (Hintzman et al., 1975). Interestingly, semantic relatedness effects appear consistently in conjunction with temporal contiguity effects in free recall experiments, and the organization of memory recall in tasks involving a semantic structure (within the encoding set and/or retrieval cues) suggests an interaction of semantic and temporal context information (Healey et al., 2019; Polyn et al., 2011). While this work highlights temporal contiguity and semantic relatedness as two key interacting factors in the organization of free recall, it is unclear whether these principles extend to memory for the temporal order of events, an important feature of episodic memory. In a study by Zöllner et al. (2022), participants exhibited a semantic clustering effect in their recall of the event order suggesting that temporal order memory is influenced by semantic associations between event features. However, whether similar effects of semantic knowledge can be shown in a task specifically designed to probe encoding and retrieval of temporal order information remains to be investigated. Following the core notion of the SCM, we expected better temporal order memory performance on trials where temporal-episodic and semantic associations between items are congruent (in that semantically related items were also encoded closer to each other in time), as compared to those where both dimensions are incongruent. This semantic congruence effect on temporal order memory is the main prediction of this current study.

In addition, it is unclear whether effects of semantic knowledge on temporal order memory would depend on a semantically structured encoding set, which may evoke semantic processing at encoding. The SCM makes predictions about the interaction of episodic memory traces and semantic information during retrieval but does not explicitly incorporate encoding processes into this proposed interplay. However, the presence of pronounced semantic structure at encoding may shift the memory system towards semantic context processing (Healey et al., 2019; Morton & Polyn, 2016). This is supported by neuroimaging results showing that neural coding of current and recent semantic category information at encoding predicts recall organization (Chan et al., 2017; Morton & Polyn, 2017). Conversely, the effect of semantic associations between encoded items on temporal order memory retrieval could be independent of semantic structure within the encoding set and primarily result from weakly encoded episodic memory and/or retrieval-specific processes as emphasized by the SCM. By specifically investigating the effect of semantically structured versus unstructured encoding sets, we aimed to establish the role of encoding processes for the interaction between episode-specific information and semantic knowledge in temporal order memory, thereby informing the SCM. We expected that participants who encoded semantically structured item sequences would show an enhanced semantic congruence effect relative to those presented with unstructured sequences.

Concerning modulating factors, the SCM would predict a more pronounced influence of semantic knowledge on memory retrieval for weakly encoded episodic memory representations as there should be an increased need for completion of the episodic gist information. Since episodic associations between items are known to be determined by the temporal lag between these items at encoding (e.g., serial positions within an encoded list) and are typically stronger for forward versus backward encoding transitions (Healey et al., 2019), these factors may modulate the influence of semantic knowledge on temporal order memory. Specifically, and following the SCM, we expected the semantic congruence effect to be more pronounced with greater temporal lag and for backward versus forward encoding transitions between items, as these factors should be associated with reduced episodic association strength and an increased need for semantic completion of the memory. In addition, increased activation of semantic concepts at retrieval should result in a more pronounced impact of semantic knowledge. Semantic typicality, which refers to the degree to which an element is representative of its associated semantic category (Rosch et al., 1976), is a relevant measure in this regard. Semantically typical items are thought to more strongly evoke categorical representations (Collins & Loftus, 1975), and location memory for such items has been shown to be influenced by semantic associations to a greater extent (Tompary & Thompson-Schill, 2021). Based on this prior work, we expected that higher semantic typicality of items presented at retrieval would be related to an increased impact of semantic associations on temporal order memory, as reflected by a stronger semantic congruence effect.

The current study aimed at investigating the impact of semantic knowledge on memory for the temporal order of event sequences. We conducted two experiments using a temporal order memory task in which participants were presented with alternating encoding and retrieval runs, consisting of encoding sequences of naturalistic visual stimuli and immediate retrieval runs involving forced-choice temporal proximity judgements. In the first experiment, encoding sequences were semantically structured such that images from the same semantic category were clustered in time. This semantic structure was not present in the second experiment where images were presented at random sequence positions. At retrieval, participants were presented with one cue image and two choice images, one of which was semantically related to the cue. Trials in which the semantically related choice was the correct one constituted congruent trials. In line with the SCM, we predicted that participants would draw on both temporal and semantic associations between encoded items during temporal order memory retrieval, resulting in better performance on congruent retrieval trials. The semantic congruence effect was predicted to be stronger for retrieval trials featuring items which were encoded in backward versus forward encoding direction and for items encoded with greater temporal lag (referred to here as encoding distance), showing the modulatory influence of episodic association strength. Greater semantic typicality of the retrieval cue was expected to be associated with a more pronounced semantic congruence effect, reflecting the modulatory effect of semantic concept activation. Finally, assuming that processing of semantic structure at encoding would increase the likelihood of temporal order memory being influenced by semantic associations, the semantic congruence effect was expected to be more pronounced in Experiment 1 compared to Experiment 2. Nevertheless, based on predictions by the SCM regarding semantic construction at retrieval, we still expected to find the semantic congruence effect in Experiment 2.

## Experiment 1

### Materials and methods

#### Participants

The required minimum sample size was estimated using G*Power 3.1 (Faul et al., 2009) and based on an expected medium effect size for the main effect of the within-subjects factor “congruence” (*d* = 0.5, *α* = 0.05, 1-*β* = 0.8, two-sided, paired). This resulted in a required sample size of 35 participants. To detect any potential effects of interest in our newly created temporal memory task, we chose to further increase the number of participants beyond this estimated minimum required sample size.

Participants were recruited through advertisements on social media networks and at the campus of Ruhr University Bochum. Inclusion criteria comprised: age ranging between 18 and 35 years, no acute neurological and psychiatric illnesses, and normal or corrected-to-normal vision. Dropout due to red-green color vision deficiency (*n* = 2) resulted in a final sample size of 54, including 39 women and 15 men aged between 18 and 35 (*M* = 22.8, *SD* = 3.0) years. While six participants completed online sessions (due to the study initially being designed as an online study but switched to lab-based testing when the online experiment hosting service became unavailable), the remaining 48 participants attended lab sessions.

The study received approval from the ethics committee of the Faculty of Psychology at Ruhr University Bochum (application number 764), following the guidelines of the Declaration of Helsinki. Participants provided written informed consent and were reimbursed with 10€ or course credits.

#### Stimulus material

Stimuli were selected from the THINGS database (Hebart et al., 2019) providing images of common object concepts from 27 high-level semantic categories. The complete image pool from which each participant-specific stimulus set was drawn consisted of 8500 images including 588 different living and non-living objects from eleven selected high-level categories: animal, clothing, electronic device, fruit/vegetable (this category was combined from the two separate THINGS categories fruit and vegetable), furniture, musical instrument, office supply, plant, sports equipment, toy, and vehicle. A unique set of images including objects from all eleven high-level categories was drawn from the pool for each participant.

Stimulus selection was pseudo-randomized as for each participant, for each block of the temporal memory task, six different categories were drawn and for each of these categories, five different objects were drawn from the complete pool of images. For each of these objects, one image was finally drawn out of the available set. Each participant-specific stimulus set comprised between 321 and 333 (*M* = 328) different images corresponding to between 226 and 264 (*M* = 246) different objects. Since each category and object could appear in more than one block for a given participant, the number of different objects and individual images slightly varied between participants. The same object could appear a maximum number of six times in different blocks, while the same individual image could appear a maximum number of three times in different blocks for a given participant. Individual color images showed single objects in scenes and were generally presented on a white background screen throughout the experiment. During encoding runs of the temporal memory task, images were displayed at 500 × 500 pixels. During retrieval runs, cue images were also presented at 500 × 500 pixels, while choice images were shown at 300 × 300 pixels.

#### Temporal memory task

**Fig. 1:**
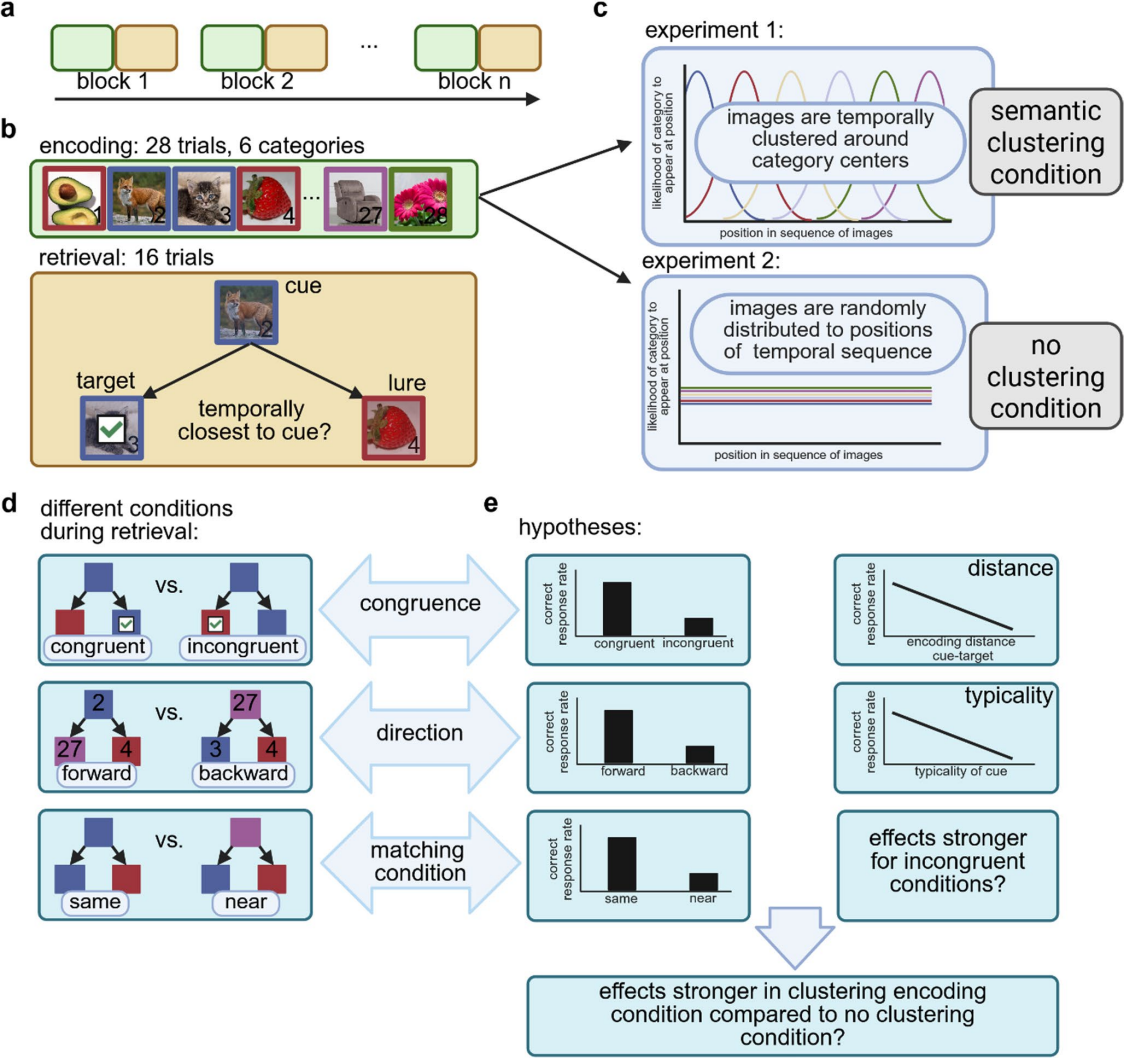
*Procedure and experimental task design* **a** The experimental task consisted of *n* blocks (*n* = 12 in Experiment 1 and *n* = 10 in Experiment 2), each including an encoding run and a retrieval run. **b** Each encoding run consisted of a sequence of 28 images, belonging to six distinct semantic categories. The images were either temporally clustered around category centers within the sequence (Experiment 1, depicted in upper section of **c**), or presented in random order (Experiment 2, depicted in lower section of **c**). Each retrieval run included 16 trials. In each trial, participants were first presented with a cue image. Subsequently, participants were presented with the cue image and two choice images. Participants were asked to indicate which of the two choice images (target = correct choice, lure = incorrect choice) was temporally closer to the cue image during encoding. **c** During encoding in Experiment 1 (semantic clustering condition), the positions of images within the sequences were not random, but clustered around category centers. That is, the probability of an image from a given category to appear was highest close to its associated category center. In contrast, in Experiment 2 (no clustering condition), all images were presented in random order. **d** During retrieval, cue and choice images were chosen based on equally distributed conditions: Trials were either congruent or incongruent; that is, either the target or the lure were semantically related to the cue image. Trials featured items either encoded in forward or in backward direction; that is, the choice images were either presented after or prior to the cue image at encoding. Finally, trials in Experiment 1 involved either same or near matching condition; that is, either target or lure belonged to the same category as the cue, or target or lure belonged to a category cluster near the category cluster of the cue. **e** Our hypotheses predicted a higher correct response rate for congruent compared to incongruent, for forward compared to backward and for same matching condition compared to near matching condition trials. Furthermore, we predicted a higher encoding distance between cue and target to be associated with a lower correct response rate and a higher typicality of the cue to be associated with a lower correct response rate. We predicted the previous effects to be stronger for incongruent trials. In analyzing data from Experiment 2 (no clustering condition) and comparing effects between experimental encoding conditions, we were interested in whether these predictions could be confirmed nonetheless, in which case semantic congruence effects could be attributed to retrieval-specific processes rather than encoding of semantic structure. The figure was created in https://BioRender.com

The temporal memory task was composed of alternating encoding runs during which participants were presented with image sequences and retrieval runs consisting of forced-choice temporal memory retrieval trials (Fig. 1). The task was divided into twelve blocks, with each block consisting of one encoding run and one subsequent retrieval run. All participants completed the same experimental task condition. The task was displayed on a computer screen at a resolution of 1920 × 1080 pixels and responses were given using a standard computer mouse.

During encoding runs, images were presented individually for two seconds in each trial for a total of 28 trials. After each image presentation, a fixation cross was displayed for 500 milliseconds. For each encoding run, six different categories were randomly drawn from the eleven available categories. From each of these six selected categories, five different objects were randomly chosen and one image was randomly drawn from the available image set for each object, resulting in 30 selected images per encoding run. The image sequences were constructed in a systematic way such that the probability of occurrence of an image from a given category was approximately normally distributed around a “category center” (i.e., category- and run-specific position within the sequence). The images were therefore temporally clustered based on their semantic category, introducing a temporal sequence of individual images and an underlying temporal sequence of image categories for each encoding run. Clusters of images were not clearly separated from each other – images from neighbouring categories could be interspersed. The first and last image of a given category within a given encoding run were on average separated by eight sequence positions. While spreads as little as 3 sequence positions and as much as 27 sequence positions between the first and last image of a category could occur due to the probabilistic approach in constructing the sequences, we confirmed that the distribution of categories along the sequences followed the intended structure. The task version used in Experiment 1 is referred to here as “semantic clustering condition”.

The first and the last item of each sequence were cut from the encoding set (resulting in 28 instead of 30 images), since otherwise these images would have virtually always belonged to the first and the last categories of the underlying sequence of category clusters, respectively, substantially reducing the variance of semantic categories at these sequence positions. The underlying sequence of categories was never the same for two or more encoding runs within one participant. Importantly, participants were not explicitly made aware of the category-based clustering of images and the consequent sequence of semantic categories underlying each encoding run.

Following encoding, immediate memory retrieval occurred after a delay of 20 s. During retrieval runs, participants performed 16 trials of forced-choice temporal memory retrieval based on the image sequence from the preceding encoding run. On each trial, participants judged the relative temporal proximity of two choice images to a cue image. The image set for each trial was pseudo-randomly selected applying constraints regarding the equal distribution of retrieval trial parameters as outlined below. A fixation cross appeared for a duration of 0,5 s before the cue image was individually displayed for one second. Two images from the encoding run were then presented immediately afterwards, and participants were prompted to select the one that appeared temporally closer to the cue item in the preceding encoding run. There was no response timeout, the retrieval trial ended as soon as the participant gave a response, after which there was a post-trial gap of one second. Feedback indicating the percentage of correct responses was presented on the screen after each retrieval run.

Retrieval trials were defined by the parameters “congruence” (congruent/incongruent), “matching condition” (same/near) and “encoding direction” (forward/backward). If the target (i.e., the correct choice image) belonged to the same category as the cue image or to the category cluster near the category cluster of the cue image, the retrieval trial was defined as “congruent”. In “incongruent” trials, the lure image (i.e., reflecting the incorrect choice) belonged to the same category as the cue image or to the category cluster near the category cluster of the cue image. Moreover, if the target or lure image was from the same category as the cue image, the matching condition of the trial was coded as “same” and if it was from a category whose cluster was near the category cluster of the cue image, it was coded as “near”. The “near” matching condition was not limited to the categories whose clusters were directly next to the cue’s category cluster, the separation could be up to four cluster positions. However, in approximately half of the “near” matching condition trials, the category was the one right next to the cue’s category. In terms of encoding direction, the target and lure images could have appeared either both after (“forward” encoding direction) or both before (“backward” encoding direction) the cue image within the encoding sequence. These parameters constrained the item set selection for retrieval trials: For each retrieval run, we ensured that there were exactly four trials of each combination of congruence and matching condition (congruent-same, congruent-near, incongruent-same, incongruent-near) as well as exactly eight “forward” encoding direction trials and eight “backward” encoding direction trials. Congruence/matching condition and encoding direction were controlled independently of each other. Item selection was implemented by first drawing a random set of three images from the encoding sequence, defining encoding direction, congruence and matching condition, then enforcing the pre-defined quotas for each trial type and finally fully shuffling the order of the resulting retrieval trial sequence. Cue-target encoding distance was not explicitly matched between the different trial conditions during the item selection process.

#### Analysis of behavioral data

Cleaning and preparation of behavioral data from the temporal memory task was performed using custom scripts in Python v3.11 (VanRossum & Drake, 2010). There were no missed responses and the complete dataset consisting of 9216 retrieval trials from 48 participants in the lab sessions was included in further analyses. The remaining 1152 trials from six participants who completed online sessions also did not include any missed responses. However, they were initially excluded from the statistical analyses due to concerns about the comparability of the different experimental settings (lab versus online). In a subsequent step, this additional data was incorporated into the analyses to evaluate if this would substantially change the pattern of results. Results from analyses of the complete dataset are reported when no substantial differences were detected.

Statistical analyses were performed using R Statistical Software (v4.3.2; R Core Team, 2023). In a first step, performance in the temporal memory task was compared against chance-level performance using a two-sided exact binomial test. Next, a random intercept logistic generalized linear mixed-effects model (GLMM) was estimated on a single-trial level to assess the effect of the five independent variables of interest on the binary dependent variable “response” (incorrect/correct) and to consider the nested trial structure in this experiment, where each participant generated 192 retrieval trials. The fixed factors in the model included the binary predictors “congruence” (congruent/incongruent), “matching condition” (same/near) and “encoding direction” (forward/backward) as well as the continuous predictors “encoding distance (cue-target)” and “cue typicality”. Note that only the encoding distance (that is, number of positions between items in the encoding sequence) between the cue and target stimuli of each retrieval trial was entered into the model as this measure strongly correlated with the encoding distance between cue and lure stimuli. In addition, we assumed that cue-target encoding distance would best reflect the critical episodic association underlying the temporal memory response on a given trial of the task. Continuous predictors were standardized before entering the model applying standard *z*-score standardization. As potential modulatory effects of the different variables on the semantic congruence effect on temporal order memory retrieval were of interest in the present study, the following two-way interactions were included in the model as fixed effects predictors: “congruence x matching condition”, “congruence x encoding direction”, “congruence x encoding distance (cue-target)” and “congruence x cue typicality”. The random factor “participant” was entered into the model to account for the nested trial structure, and random intercepts as well as random slopes for the individual within-subject predictors were modeled. The GLMM used a logit link function and was fit using maximum likelihood estimation as provided by the “glmer” function of the R package lme4 (Bates et al., 2015). Odds ratios (*OR*) and 95% confidence intervals (*CI*) are reported as indicators of statistical significance for each fixed effect predictor. In the case of logistic GLMMs, a confidence interval of OR which does not include the value 1 indicates that a given predictor is statistically significant (Faraway, 2016).

### Results

**Fig. 2:**
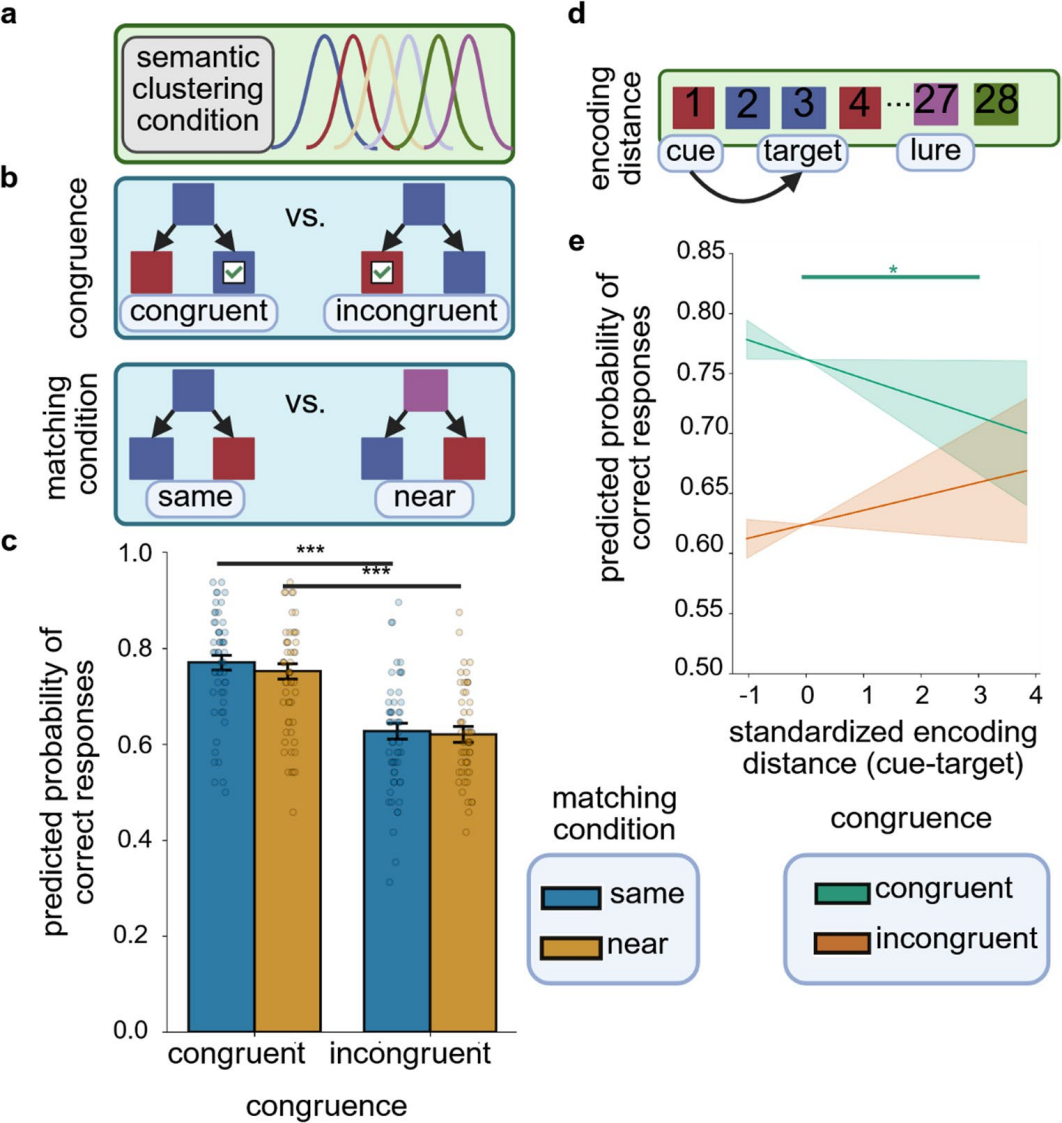
*Temporal order memory performance in Experiment 1* **a** In Experiment 1, the probability of an image to appear at a given position within the sequence of images was determined by the proximity to the category center position of the respective semantic category. **b** Congruence was determined by semantic associations between the cue and choice images, whereby on congruent trials, cue and target (the correct option) were semantically related (i.e., they belonged to the same category or to neighboring category clusters with respect to the encoding sequence) and on incongruent trials, cue and lure (the incorrect option) were semantically related. Matching condition was defined by the type of semantic relatedness between cue and target or cue and lure, whereby on same condition trials, the semantically related items belonged to the same category and on near condition trials, the semantically related items belonged to category clusters which had neighboring center positions within the encoding sequence. **c** The probability of correct responses was significantly predicted by congruence, whereby congruent trials had a higher probability of correct responses compared to incongruent trials. This effect did not differ between matching conditions. Colored bars show model estimates, error bars depict standard errors of estimated means and data points show mean hit rates for individual participants. **d** Encoding distance was defined as the standardized temporal distance between cue and target (the correct choice) during encoding, that is, the number of positions between both images within the sequence of images. **e** There was a significant two-way interaction between congruence and encoding distance. Post-hoc analyses showed that encoding distance was a significant negative predictor of correct responses only on congruent trials. In addition, the benefit of congruent over incongruent trials was larger for smaller encoding distances. Plotted lines show model estimates and shaded areas depict 95% confidence intervals for estimated trends. *** *p* < 0.001; * *p* < 0.05. The figure was created in https://BioRender.com

As revealed by the exact binomial test of the ratio of correct trials against a chance level of 0.5, participants performed significantly above chance in the temporal memory task (*P*_correct response_=0.68, *p* < 0.001). This was likewise true for the complete dataset including data from the online sessions (*P*_correct response_=0.68, *p* < 0.001). The general result pattern of the logistic GLMM analysis did not change after including data from the online sessions, therefore only results from the analysis of the complete dataset will be reported hereafter. The logistic GLMM analysis revealed a significant main effect of congruence on responses in the temporal memory task. Participants were significantly more likely to give a correct response on congruent as compared to incongruent retrieval trials (*OR* = 1.87, 95% *CI* (*OR*) [1.52–2.29], Fig. 2). None of the remaining variables were significant individual predictors of correct responses (all 95% *CI* (*OR*) [< 1,>1]). In addition, the interaction between congruence and encoding distance was statistically significant (*OR* = 0.87, 95% *CI* (*OR*) [0.79–0.97], Fig. 2). Post-hoc comparisons of estimated trends showed that cue-target encoding distance was significantly negatively associated with correct responses on congruent trials (*β*=−0.016, *SE* = 0.008, *p* = 0.046), but not on incongruent trials (*β* = 0.012, *SE* = 0.008, *p* = 0.146). With lower cue-target encoding distance, the benefit on congruent versus incongruent trials became larger (at encoding distance = −1: *OR* = 2.21, 95% *CI*_*Tukey*_ (*OR*) [1.83–2.66]; at encoding distance = 1: *OR* = 1.68, 95% *CI*_*Tukey*_ (*OR*) [1.39–2.02]).

### Discussion

Experiment 1 used semantically structured sequences at encoding and probed effects of semantic knowledge on memory for the relative temporal order of events. Results showed that, while participants exhibited above-chance level retrieval performance, there was a significant benefit of semantically congruent over incongruent retrieval trials. That is, temporal order memory performance was better when semantic and temporal associations between items would have indicated the same response compared to when semantic and temporal associations were at odds. We observed that this effect emerged when semantic associations were established by same-category items as well as when items came from neighboring category clusters of the encoding sequence. This latter finding suggests that the semantic structure of the encoding sequence may give rise to the observed semantic congruence effect. To test this possibility, we conducted a second experiment which used encoding sequences that involved items from different categories being presented at random sequence positions, thereby omitting pronounced sematic structure.

## Experiment 2

### Materials and methods

#### Participants

In Experiment 2, participants were recruited in the same manner as in Experiment 1, the same inclusion criteria were applied and participants were compensated in the same way. Due to red-green color vision deficiency one participant had to be excluded. The remaining 35 participants (24 women, 10 men, 1 diverse) were between 18 and 35 (*M* = 24.9, *SD* = 4.6) years old. All participants completed the experimental sessions in the lab.

#### Stimulus material

For Experiment 2, the stimuli were selected from the same database and in the same manner as in Experiment 1. Here, due to the number of blocks in the temporal memory task being reduced to ten, each participant-specific stimulus set comprised 280 different images corresponding to between 214 and 236 (*M* = 226) different objects. Similar to Experiment 1, because each category and object could appear in more than one block for a given participant, the number of different objects slightly varied between participants. The same object could appear a maximum number of two times in different blocks, while the same individual image could appear only once across all blocks for a given participant.

#### Temporal memory task

In Experiment 2, a slightly modified version of the experimental task was used. Participants were asked to complete ten blocks instead of twelve, a modification which was made to adapt and test the experimental task for later use in an fMRI environment. In this version of the temporal memory task (referred to here as “no clustering condition”), images were presented without any clustering based on their semantic category. Instead in any given encoding run, images from all six categories appeared at random sequence positions. As in Experiment 1, all participants in this experiment completed the same task condition.

During encoding runs, stimuli were presented for two seconds after which a fixation cross was displayed for a randomly chosen duration between 1,5 and 2 s (this was again implemented for testing of an fMRI-adapted task version). During retrieval runs, a fixation cross was shown for a randomly chosen duration between 1,5 and 2 s before the cue image for a given retrieval trial was individually presented for 2 s. This was followed by a fixation cross that was displayed for a randomly chosen duration between 0,5 and 1 s. The two choice images were presented subsequently with a prompt asking the participant to select the one that appeared temporally closer to the cue image in the preceding encoding run. There was a response timeout of 4,5 s. The retrieval trial ended as soon as the participant gave a response.

The variable “congruence” had three levels in this task version (congruent/incongruent/categories unrelated). In trials labelled as “categories unrelated”, the choice images both belonged to a different category than the cue. Importantly, as there was no underlying sequence of category clusters in encoding runs of Experiment 2 due to the omission of category-based clustering, the variable “matching condition” was obsolete here as neighboring categories were not possible. As in Experiment 1, retrieval trial parameters constrained the item selection, however the quotas for each trial type differed in comparison to Experiment 1 due to the absence of the variable matching condition. In each retrieval run, there were exactly four congruent and four incongruent trials, with the target (congruent trials) or lure (incongruent trials) being from the same category as the cue on each one. In addition, there were eight “categories unrelated” trials which featured images from three different categories. As in Experiment 1, there were exactly eight “forward” and eight “backward” encoding direction trials, which was controlled for independently of the remaining parameters. After a sequence of 16 retrieval trials had been built applying the above constraints, the trial order was fully shuffled for each retrieval run. Cue-target encoding distance was not explicitly matched between the different trial conditions during the item selection process.

#### Analysis of behavioral data

For Experiment 2, the same data cleaning and preparation procedure as in Experiment 1 was applied, with the added removal of 48 retrieval trials where participants failed to respond within the time limit. This resulted in a dataset including 5552 retrieval trials from 35 participants. Again, performance in the temporal memory task was first compared against chance-level performance using a two-sided exact binomial test. Moreover, a similar GLMM as in Experiment 1 with “response” (incorrect/correct) as dependent variable was estimated, however in this case, the predictor “congruence” had three levels (incongruent/congruent/categories unrelated). The last level applied to retrieval trials in the no clustering condition in which all three stimuli (cue, target, lure) belonged to different categories. The three levels were represented by two binary dummy variables in the model. The remaining fixed effects predictors that were also included in the GLMM analysis for Experiment 1 (“encoding direction”, “encoding distance (cue-target)” and “cue typicality”) were all entered into the model, as well as the two-way interactions between each of these predictors and the predictor “congruence”. Again, the random factor “participant” was included to account for the nested trial structure, modelling random intercepts and random slopes for the individual within-subject predictors.

### Results

**Fig. 3:**
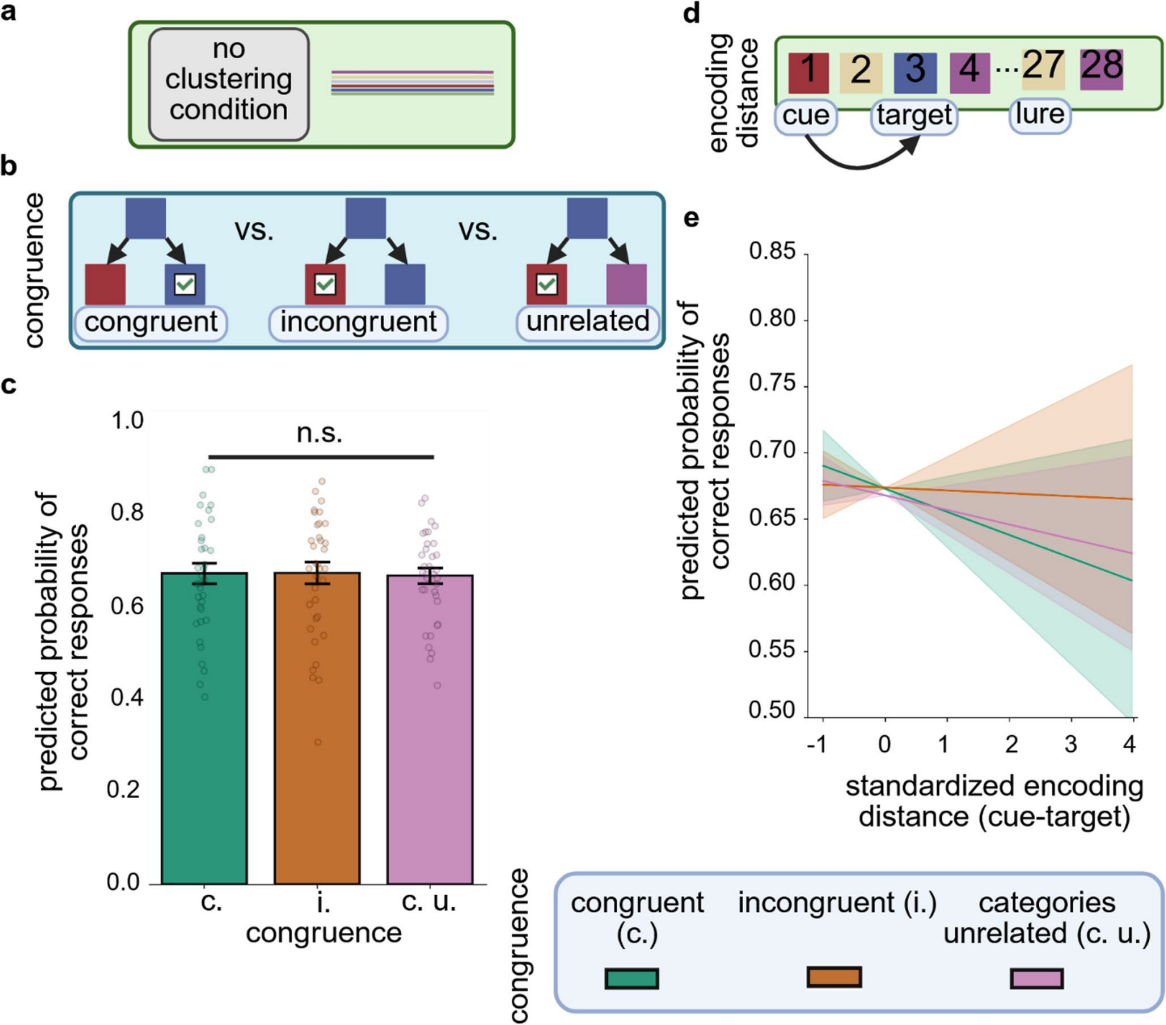
*Temporal order memory performance in Experiment 2* **a** In Experiment 2, the probability of an image to appear at a given position within the sequence of images was random. **b** Congruence was determined by semantic associations between the cue and choice images, whereby on congruent trials, cue and target (the correct option) were semantically related (i.e., they belonged to the same category) and on incongruent trials, cue and lure (the incorrect option) were semantically related. On categories unrelated trials, neither target nor lure was from the same category as the cue. **c** The probability of correct responses did not differ significantly across the three levels of congruence. Colored bars show model estimates, error bars depict standard errors of estimated means and data points show mean hit rates for individual subjects. **d** Encoding distance was defined as the standardized temporal distance between cue and target (the correct option) during encoding, that is, the number of positions between both images within the sequence of images. **e** Encoding distance between cue and target did not significantly predict the probability of correct responses. For reasons of comparability with Experiment 1, results are plotted separately for congruent, incongruent and categories unrelated trials. Lines show model estimates and shaded areas depict 95% confidence intervals for estimated trends. n.s. = not significant. The figure was created in https://BioRender.com

As in Experiment 1, participants performed significantly above chance level in the temporal memory task (*P*_correct response_=0.66, *p* < 0.001). The logistic GLMM analysis revealed no significant main effects or interactions (all 95% *CI* (*OR*) [< 1,>1], Fig. 3).

### Discussion

In contrast to Experiment 1, which used semantically structured encoding sequences, Experiment 2 used encoding sequences that had images from different categories appearing at random sequence positions. While participants again showed above-chance level retrieval performance, the semantic congruence effect that was observed in Experiment 1 was not found in Experiment 2. This indicates that semantic structure at encoding may have driven this effect. In order to compare both datasets more directly, we next conducted a joint analysis across Experiments 1 and 2.

## Joint analysis of Temporal memory task data from experiments 1 and 2

In order to directly compare temporal order memory effects between Experiment 1 (semantic clustering condition) and Experiment 2 (no clustering condition), data from both experiments were analyzed using a single model. To this end, retrieval trials with the congruence level “categories unrelated” were excluded from the dataset of Experiment 2, since this type of retrieval trial did not exist in Experiment 1. The resulting 13,139 retrieval trials from 89 participants were included in the statistical analysis. Fixed effects predictors in the logistic GLMM included “encoding condition” (semantic clustering condition/no clustering condition), “congruence” (incongruent/congruent), “encoding direction”, “encoding distance (cue-target)” and “cue typicality”. Three-way interactions between “encoding condition”, “congruence” and each of the remaining fixed effects predictors were entered into the model as well as the random factor “participant” modeling random intercepts and random slopes for the individual within-subject predictors.

## Results

**Fig. 4:**
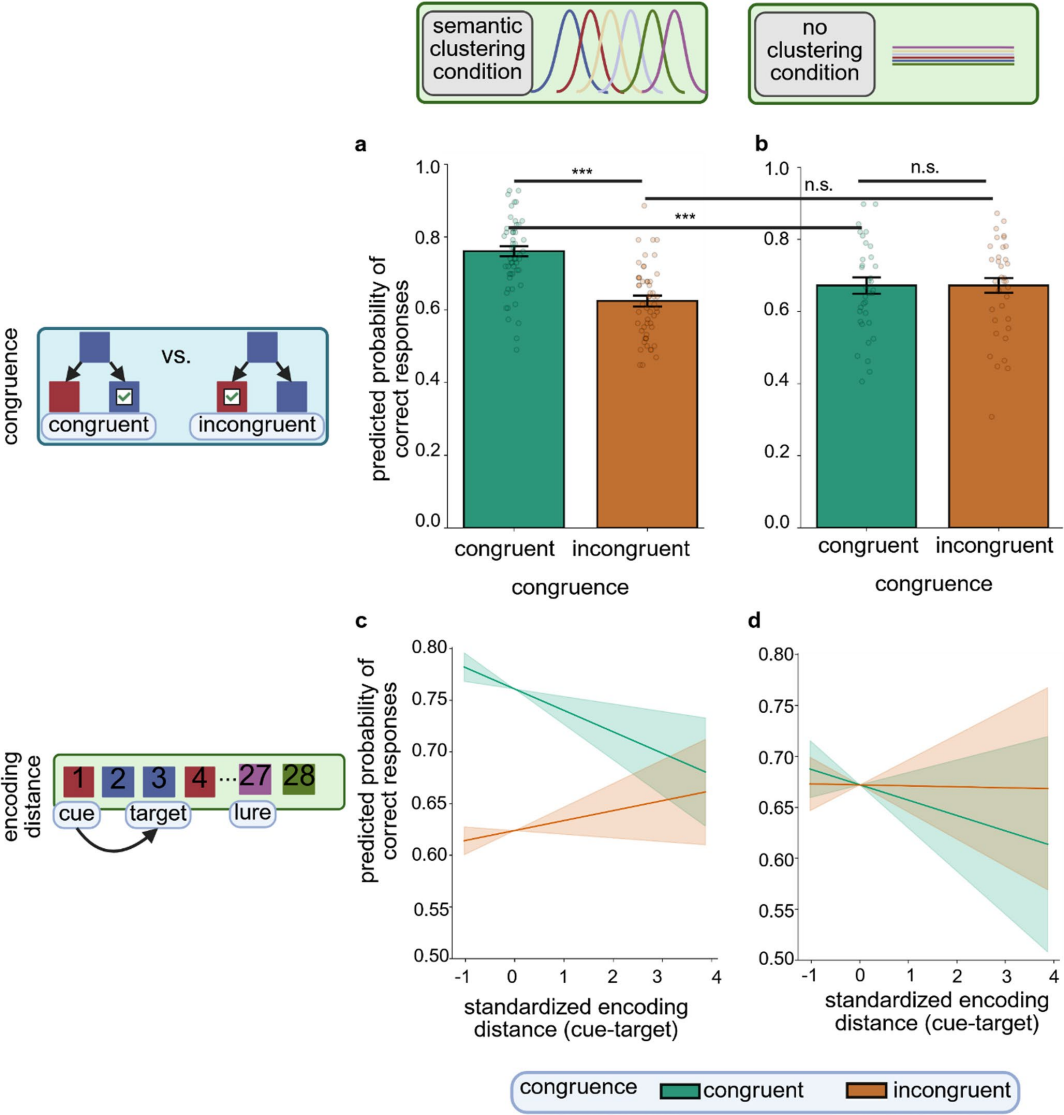
*Temporal order memory task performance across Experiments 1 and 2* The predicted probability of correct responses for congruence (**a** and **b**) and encoding distance between cue and target (**c** and **d**) was estimated for the semantic clustering condition (**a** and **c**) and the no clustering condition (**b** and **d**). In **a** and **b**, colored bars show model estimates, error bars depict standard errors of estimated means and data points show mean hit rates for individual participants. In **c** and **d**, lines show model estimates and shaded areas depict 95% confidence intervals for estimated trends. We found an interaction effect of encoding condition and congruence. Post-hoc analyses revealed a significantly higher probability of correct responses for congruent compared to incongruent trials in the semantic clustering condition and a significantly higher probability of correct responses for congruent trials in the semantic clustering condition compared to congruent trials in the no clustering condition. We did not find a significant main effect or interaction of cue-target encoding distance in the joint analysis of both datasets. *** *p* < 0.001; n.s. = not significant. The figure was created in https://BioRender.com

The GLMM which was fit to the complete dataset showed a significant two-way interaction between congruence and encoding condition (*OR* = 1.88, 95% *CI* (*OR*) [1.36–2.60], Fig. 4). As per post-hoc comparisons of estimated marginal means, participants were significantly more likely to give a correct response on congruent as compared to incongruent trials in the semantic clustering condition (that is, in retrieval trials of Experiment 1; *OR* = 1.92, 95% *CI*_*Tukey*_ (*OR*) [1.65–2.23]; cf. Results of Experiment 1), while this contrast was not statistically significant in the no clustering condition (that is, in retrieval trials of Experiment 2; *OR* = 1.00, 95% *CI*_*Tukey*_ (*OR*) [0.80–1.25] cf. Results of Experiment 2). In addition, participants who completed the semantic clustering condition of the task (Experiment 1) showed significantly better performance than participants who completed the no clustering condition (Experiment 2) on congruent trials (*OR* = 1.55, 95% *CI*_*Tukey*_ (*OR*) [1.21–1.99]). The contrast between semantic clustering condition and no clustering condition was not significant for incongruent trials (*OR* = 0.81, 95% *CI*_*Tukey*_ (*OR*) [0.65–1.01]).

## Discussion

This study investigated how semantic knowledge influences memory for the temporal order of events. We found that when image sequences were semantically clustered, temporal order memory benefited on congruent retrieval trials (cue and target semantically related) compared to incongruent trials (cue and lure semantically related). This semantic congruence effect occurred when semantically related items belonged to the same category as well as when related items belonged to categories with neighboring clusters in Experiment 1. The effect was absent in Experiment 2, where encoding sequences lacked semantic clustering. A combined analysis confirmed these results. Moreover, in Experiment 1, cue-target encoding distance negatively predicted accuracy on congruent trials, and the congruence effect was stronger with smaller encoding distances. These findings suggest participants used semantic context to support temporal memory, but only when semantic structure was present during encoding. The benefit for congruent over incongruent trials likely reflects alignment between semantic and temporal associations during encoding.

Our results align with prior work showing that semantic associations influence episodic memory across tasks, including free recall (Aka et al., 2021), recognition (Montefinese et al., 2015), and spatial and temporal memory (Lu et al., 2024; Tompary & Thompson-Schill, 2021; Ishiguro & Saito, 2021). Notably, participants in these studies, as in ours, were not informed of the underlying semantic structure, indicating that effects arose from activation of pre-existing semantic knowledge.

The congruence effect in Experiment 1 extended to ‘near’ category trials, suggesting that retrieval was guided by the encoded semantic context rather than by semantic cueing at retrieval alone. Moreover, the finding that encoding distance between cue and target negatively predicted correct responses specifically in the congruent condition of Experiment 1 indicates that the congruence effect was not merely driven by a heuristic (e.g., always choosing the same-category item when available). If such a heuristic had been used, encoding distance should not have influenced performance on congruent trials. Finally, the absence of a congruence effect in Experiment 2 further supports the conclusion that encoding of semantic context drives the benefit for congruent over incongruent trials in the semantic clustering condition. Prior studies similarly show that effects of semantic relatedness on memory emerge only when semantically structured item sets are encoded (Kowialiewski et al., 2021, 2022; Melega & Sheldon, 2023; Aka et al., 2021). These findings support models like the Context Maintenance and Retrieval model (CMR; Polyn et al., 2009), which posits that during encoding, the activation of pre-established semantic associations feeds into an active context representation that subsequently guides retrieval. The current study suggests that the underlying mechanisms extend to memory for the temporal order of events. Importantly, while the semantic congruence effect observed in the present study is generally in line with predictions made by the Scenario Construction Model, the fact that this pattern was only found in the semantic clustering encoding condition implies that encoding processes, rather than generative retrieval mechanisms alone, contributed to this effect, as discussed furhter below.

Whether semantically structured versus unstructured encoding broadly benefits temporal order memory remains an open question. Differences in data collection across Experiments 1 and 2 caution against direct comparison of overall accuracy. However, our joint analysis suggests that benefits may be confined to congruent trials under structured encoding. While some meta-analyses report increased errors with semantic structure in temporal tasks, especially those that do not involve individual item retrieval (Ishiguro & Saito, 2021), others show benefits when clustering is present (Kowialiewski et al., 2024). Conversely, semantic relatedness impaired location memory in a design where all items shared one category but lacked category-specific structure (Lu et al., 2024). Moreover, recent evidence from free recall experiments and simulations shows that strong semantic structure of a study list can disrupt the temporal organization of memory recall, especially when semantic and temporal structure of the list are not in line with each other (Hong et al., 2024). Thus, while not necessarily detrimental to free recall performance overall, semantic context encoding may be beneficial to spatial or temporal memory only when it aligns to some degree with spatial or temporal structure at encoding and therefore provides a more effective organizational factor for memory. The Scenario Construction Model (Cheng et al., 2016), which primarily emphasizes retrieval mechanisms and makes few explicit assumptions about encoding processes, may be informed by the present findings. Specifically, we suggest that the SCM could be extended to incorporate the encoding of semantic context as an important factor determining the relative contributions of episode-specific information and semantic knowledge to subsequent memory retrieval. Such semantic encoding processes, in interaction with factors such as the overall strength of the episodic memory trace and retrieval-specific mechanisms, may bias the memory system toward encoding semantic associations between elements of an episode more strongly, thereby increasing the influence of semantic knowledge on later memory for episodic (temporal, spatial) aspects of that episode.

We predicted that the semantic congruence effect would be stronger when cue-target pairs were encoded with greater temporal distance and for backward versus forward encoding direction trials. Our results showed that encoding distance negatively predicted accuracy on congruent trials in Experiment 1 and – against our hypothesis – that the congruence effect was more pronounced with smaller encoding distances. The absence of this interaction in Experiment 2 suggests that encoding distance effects were related to the way in which semantically clustered item sequences were encoded in Experiment 1. These findings align with work showing that context boundaries – potentially akin to cluster boundaries in our task – affect item associability and temporal memory (DuBrow & Davachi, 2014). In Experiment 1, smaller encoding distances presumably facilitated encoding of semantic associations as items were more likely to be close to their category clusters, enhancing the congruence effect. Greater encoding distance may have reduced semantic associability between items due to intervening category clusters, which could explain the reduced congruence effect and the reduced accuracy on congruent trials with increased cue-target encoding distance in Experiment 1. In contrast, item strength effects which have been suggested to drive a beneficial impact of greater encoding distance on temporal recency judgements (Sheldon, 2021) seem less relevant here, as participants could not simply rely on selecting the item that was more strongly represented in memory. This strategy would yield correct responses in only half of the trials (i.e., those with a ‘backward’ encoding direction), potentially impairing performance. Based on our results, we cannot rule out that the strength of episodic associations – for which encoding distance served as a proxy in this study – can be a modulating factor for the impact of semantic knowledge on temporal order memory as would be expected following the SCM. Future studies should specifically manipulate episodic association strength to investigate this possibility.

We found no effect of encoding direction on temporal order memory performance. While we expected backward associations to impair performance especially on incongruent trials, previous studies have also reported no significant differences between forward and backward semantic links (Saint-Aubin et al., 2014) or transitions (Dougherty et al., 2023) in serial recall tasks. Encoding direction effects may be less reliable than previously assumed, particularly in tasks where cue items can serve as anchors for reconstructing temporal context. Whether factors linked to episodic association strength (encoding distance, encoding direction) contribute to the observed semantic congruence effect – potentially via retrieval-based completion of weak episodic traces as implied by the SCM – remains to be shown directly. Further studies are needed to explore interactions between semantic knowledge and episodic association strength.

Contrary to our hypothesis, item typicality at retrieval did not modulate the semantic congruence effect. Though prior work finds that item-specific typicality modulates the effect of semantic associations on spatial memory reconstruction (Tompary & Thompson-Schill, 2021; Tompary et al., 2023), our paradigm – involving concurrent processing of multiple items per trial and presumably, concurrent category representations – may have reduced the impact of individual item typicality. Additionally, the congruence effect here may stem more from encoding of semantic context information than from retrieval-driven semantic activation.

Some limitations apply to the current study. Data collection was split into two separate phases which may limit the validity of direct comparisons between the datasets of Experiments 1 and 2. In addition, part of the data of Experiment 1 was obtained in an online setting while most testing sessions were conducted at the lab. However, excluding online sessions did not alter results. Moreover, the analyses reported here may lack sufficient statistical power to detect certain effects of interest. Because the sample size was determined under the assumption of a moderate effect size for the main effect of congruence, it may have been too small to detect more subtle effects, such as those related to encoding direction (forward vs. backward) and typicality. Therefore, the absence of evidence for these effects should be interpreted with caution. Lastly, the experimental task used in the current study probed memory for the temporal order of events, but the reconstructive character of the memory retrieval process in this task is arguably limited, as recall of temporal order is focused on a small, predefined subset of items on every trial. While this limits the generalizability of our findings towards generative episodic retrieval processes more generally, this paradigm allows for a highly controlled examination of the effects of semantic knowledge on temporal order memory on the level of individual trials. This property may be beneficial for future neuroimaging studies investigating the neural mechanisms underlying the effects of semantic knowledge on temporal episodic memory.

## Conclusion and future outlook

This study highlights the conditions under which semantic knowledge shapes memory for the temporal order of events. Specifically, a semantic congruence effect on temporal order memory emerged only when image sequences were semantically structured at encoding, and not when images were presented in random order. Our findings suggest that the encoding of semantic context information influences temporal order memory when semantic and temporal structure of an event sequence are intertwined. These results extend prior research on the effects of semantic knowledge on various memory tasks, specifically by demonstrating the dependence of the semantic congruence effect on the encoding of semantically structured material and by extending this research to memory for the temporal order of naturalistic visual stimuli. Our findings are largely in line with predictions made by influential theoretical frameworks such as the CMR and the SCM. In addition, we provide evidence suggesting that the SCM should specifically incorporate semantic encoding processes to explain how semantic knowledge can influence generative episodic memory. Future research should explore the generalizability of the current findings across different populations and stimulus types, including more complex and naturalistic visual and spatiotemporal materials. Finally, given the potential for semantic knowledge to influence memory retrieval, understanding how different encoding strategies can affect the balance between semantic and episodic memory expression may inform interventions for disorders of memory function and applications in educational settings.

## Data Availability

Raw data and analysis scripts for Experiment 1, Experiment 2 and the joint analysis of both datasets can be retrieved from the Open Science Framework project repository using the following link: https://osf.io/26avd/.
